# The friendships of children and youth with attention-deficit hyperactivity disorder: A systematic review

**DOI:** 10.1371/journal.pone.0289539

**Published:** 2023-08-07

**Authors:** Katherine Spender, Yu-Wei Ryan Chen, Sarah Wilkes-Gillan, Lauren Parsons, Alycia Cantrill, Megan Simon, Abbygale Garcia, Reinie Cordier

**Affiliations:** 1 School of Health Sciences, Faculty of Medicine and Health, The University of Sydney, Sydney, New South Wales, Australia; 2 Curtin School of Allied Health, Faculty of Health Sciences, Curtin University, Perth, Western Australia, Australia; 3 Department of Social Work, Education and Community Wellbeing, Faculty of Health and Life Sciences, University of Northumbria, Newcastle upon Tyne, United Kingdom; University of Miami Miller School of Medicine: University of Miami School of Medicine, UNITED STATES

## Abstract

**Background:**

Children with attention-deficit hyperactivity disorder (ADHD) experience substantial difficulty maintaining meaningful friendships, which has implications for social functioning and mental health. No systematic review has investigated their friendship difficulties.

**Objectives:**

To systematically review and methodologically appraise the quality of existing studies reporting on friendships of children with ADHD. To compare their friendships to typically-developing children, and examine associations between friendship and children’s social-emotional wellbeing and mental health.

**Method:**

Six databases were searched. The methodological quality of studies was assessed using the QualSyst appraisal tool and the Appraisal tool for Cross-Sectional Studies. Aspects of friendships measured were charted, along with comparisons between children with ADHD and typically-developing children and the associations between friendships and social-emotional wellbeing and mental health.

**Results:**

Twenty-three cross-sectional studies and one longitudinal follow-up study were included. Studies included 1509 participants with ADHD, with 1197 typically-developing participants used as a companion in 19 of the 24 studies. Friendship quantity was the most investigated aspect of friendship. Children and youth with ADHD had significantly fewer friends, lower quality friendships and poorer friendship interactions. There were mixed findings from studies investigating the role or impact of friendship on social-emotional wellbeing and mental health. Twenty-two had strong methodological quality.

**Conclusion:**

Limited longitudinal studies, small sample sizes and variability in measurement restrict the interpretations of friendship over time and the causal impact of friendship on social and emotional outcomes. Further research should investigate the role and impact of friendships on the social-emotional wellbeing of children and youth with ADHD.

## Introduction

Attention-Deficit Hyperactivity Disorder (ADHD) is characterised by persistent heightened levels of inattention, and/or hyperactivity-impulsivity that significantly hinder development [1]. ADHD is the most common neurodevelopmental disorder, with global prevalence rates of 5.9% to 7.1% in children and youth [2, 3]. ADHD has many common comorbidities including oppositional defiant disorder, conduct disorder, learning difficulties and internalising disorders [1]. As a result, children with ADHD experience significant differences in social functioning in comparison to their typically-developing (TD) peers, as demonstrated by multiple systematic reviews [4, 5]. Across the literature, social functioning is considered an overarching construct encompassing three interconnected yet distinct areas: social skills, social cognition, and peer functioning [4–7]. In a recent meta-analysis conducted by Ros and Graziano [7], results from 109 studies found that within social functioning, children with ADHD had most difficulty with peer functioning, which includes peer status and friendships. The purpose of this study is to conduct a systematic review on the friendships of children and adolescents with ADHD.

### Peer functioning

Peer status is the degree to which an individual is accepted or rejected by their peers [8]. Two systematic reviews focused more broadly on peer functioning, in which 14 studies collectively demonstrated children and youth with ADHD were more likely to be peer rejected than TD peers [4, 5, 9]. The implications of peer rejection for children and youth with ADHD were an increased risk of academic failure, school dropout, depression, anxiety, substance and/or alcohol misuse [10, 11]. Furthermore, children and adolescents with ADHD are more likely to self-report experiencing peer victimisation from their peers than TD peers [12–14]. As many as 57% of adolescents with ADHD reported that they experienced any form of peer victimization at least once per week [12]. However, parents and teachers also reported that children with ADHD were more likely to be bullies themselves or threatened other peers [13]. Adolescents who had lower satisfaction with family relationships and higher scores on the Behaviour Approach Scale were significantly associated with bullying perpetration [15], yet while female adolescents with ADHD were reported to be more likely to engage in bullying than their female TD peers, this was not a significant finding [14].

Friendship is distinctly different to peer status and is defined as a close relationship between two children that is mutual, reciprocal, and voluntary [8, 16]. Through friendship, children and youth learn how to cooperate, manage conflict, and express their emotions appropriately [17–21]. Friendship has been measured most commonly by peer nominations, parent, teacher and self-report questionnaires, and observation in structured and free play tasks [21–25]. Using these types of measures, friendship has been associated with successful adjustment during stressful periods for children and youth [16].

### Conceptual models of friendship

**Fig 1:**
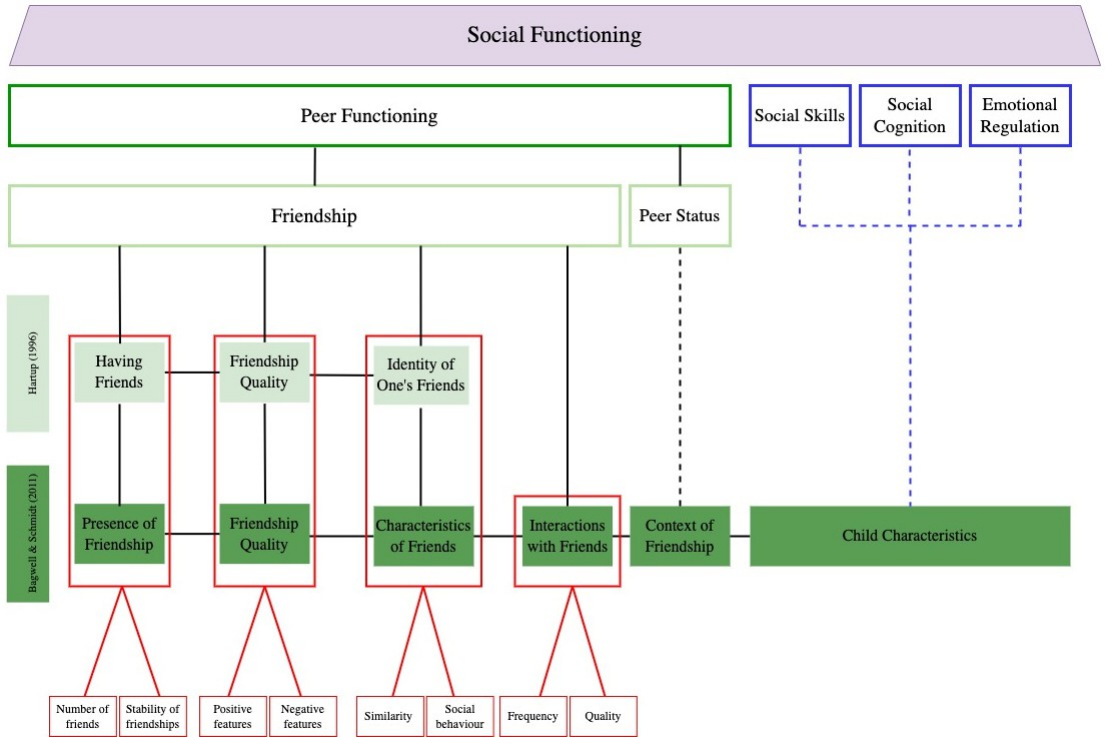
A conceptualization of friendship based on existing literature. *Note*. The conceptualisation of friendship has been adapted from both Hartup’s model of friendship and Bagwell & Schmidt’s model of friendship experience [16, 21].

With the existence of varying definitions of friendship and measures to operationalise these definitions, two prominent conceptual models of friendship have been presented within the literature [16, 21]. Fig 1 is a visual representation of social functioning that was developed by the authors to display how friendships has been operationalised in this current study and how it differs from other social functioning concepts. As friendship is multi-faceted, Hartup [16] proposed three domains of friendship: *having friends*, *friendship quality* and the *identity of one’s friends* which is represented by the light green boxes in Fig 1. Bagwell and Schmidt [21] conceptualised six domains (which is indicated in dark green in Fig 1) in their model of friendship experience, with three of their six domains (*presence of friendship*, *friendship quality* and *the characteristics of friends*) largely overlapping with Hartup [16]. The additional domains for Bagwell and Schmidt [21] were: *interactions with friends*, *child characteristics* (i.e., social skills, behaviours and competence, social cognition, emotional regulation), and *the context of friendship* (i.e., peer status). However, the inclusion of *child characteristics* and *the context of friendship* as domains of friendship contrasts with most existing literature [4, 5, 7, 9]. Multiple systematic reviews and one meta-analysis have conceptualised *child characteristics* with social functioning and *context of friendship* with peer status [4, 5, 7]. This distinction in the literature demonstrates recognition that *child characteristics* and *peer status* are separate constructs that rather contribute to a successful or unsuccessful friendship [4, 5, 7]. This distinction has been presented in Fig 1.

Therefore, we adopted a conceptualisation of friendship that reflected existing literature and included the following four domains: *presence of friendship*, *friendship quality*, *characteristics of friends* and *friendship interactions* which is indicated by the red boxes in Fig 1. The domain of *presence of friends* encompasses whether a child has a friend, the number of their reciprocated friends and the stability of their friendships (i.e. duration) [21]. *Friendship quality* is comprised of both positive features (i.e., companionship, support) and negative features (i.e., conflict) [26]. Under the domain of *characteristics of friends*, aspects may include understanding if friends share similar attitudes or interests and the social behaviours and competencies of friends [16, 21]. The domain of *friendship interactions* includes both the frequency of contact with friends and the quality of friendship interactions [21]. The quality of *friendship interactions* is distinct from *friendship quality* as it captures behaviour in games (e.g., compliance with rules), affect with friends, and how children make proposals [24, 27], rather than assessing the overall positive and negative features of a friendship [26]. Fig 1 provides a schematic representation of our conceptualisation of friendship based on existing literature.

### Existing reviews on the friendships of children with ADHD

Two previous systematic reviews on peer functioning for children and youth with ADHD, included friendship as a variable [4, 5]. Although most of the 33 studies included in these reviews investigated peer status, only six of the studies examined friendship outcomes. Girls with ADHD were found to have fewer friends and friendships that were less stable than TD girls. The results of these studies of friendship included in these reviews did not show a significant correlation between externalising symptoms and comorbid oppositional behaviour or conduct disorder with friendship outcomes [4, 5]. Of the six studies already included in systematic reviews, the most recent was published in 2011 and numerous studies assessing friendship have since been published. Four literature reviews have investigated the friendships of children with ADHD [8, 17, 28, 29]. These literature reviews lacked the methodological rigour associated with systematic reviews and neither the systematic nor literature reviews assessed the methodological quality of their included studies. Collectively, this presents a substantial gap in the literature as the friendships of children with ADHD have not been synthesized or critiqued in over a decade.

### Possible contributors to friendship challenges: Social skills, social cognition and emotional regulation

Children and youth with ADHD experience a breadth of friendship difficulties. The *presence of friendships* for children with ADHD were found to be fewer and shorter compared to TD children [30–32], with children self-reporting poorer *friendship quality*, with less positive features and more negative features [27, 30, 32–34]. Possible contributors to their poorer friendships may be difficulties with their social skills, social cognition, and emotional regulation abilities. While the distinction between social skills and friendships has been established, the development of these skills are crucial to enable successful peer relationships [7, 21]. Social skills include both verbal and non-verbal behaviours that support peer interactions such as appropriate facial expressions, sharing, helping others and turn taking [7]. The core symptoms of ADHD being inattentiveness and/or hyperactivity and impulsivity often impact the performance of these desired social skills within interactions such as interrupting others when they are talking, making irrelevant comments, increased conflict, [35–37].

Secondly, children and youth with ADHD have impaired social cognition which may also contribute to their poorer friendships. Social-cognitive skills include identifying and interpreting cues, perspective taking, forethought, cognitive biases, and self-perception of one’s performance [9, 35, 38]. Children’s difficulty with inter-personal empathy [39] could explain poorer quality interactions with friends, as cooperative play, games with rules, and successfully supporting another’s needs in play require perspective-taking. As children with ADHD have difficulty anticipating the impact of their behaviour on their friend’s emotional state and adjusting their actions accordingly, they may have fewer and shorter reciprocal friendships and develop friendships with children who have similar difficulties [35, 39, 40]. Children and youth with ADHD have reported impaired social informational processing which impacts their ability to respond to social situations appropriately [38]. Children and youth with ADHD may misinterpret social cues and can be more likely to respond to events with aggressive behaviour in neutral situations, known as a hostile attribution bias [41]. When children act defensively to perceived negative behaviour from peers, this can hinder the development of friendships [41]. Additionally, children and youth with ADHD may also hold a positive illusionary bias in which they perceive their own competence of their skills (i.e. social skills) better than their actual competence [42]. This presents a barrier to intervention if children are unable to identify areas of their own poorer social skills within their friendships.

A third explanation for their poorer friendships may be due to difficulties with emotional regulation [35, 43]. For children with ADHD, applying Barkley’s model of behaviour inhibition, highlights how it may be difficult for children to independently self-regulate [35]. Both children and youth with ADHD can struggle to self-regulate when they feel negative emotions (such as anger, frustration) and positive emotions (such as excitement) which this impacts their ability to demonstrate the desired behaviour in the social situation (i.e., staying calm after losing a game, using appropriate observable facial expressions and their tone of voice) [43, 44]. Their behaviour may be perceived as immature, bothersome or overly exuberant, in turn impacting their friendship interactions [45]. Further, difficulties with emotional regulation have been associated with more frequent incidents of verbal or physical aggression in children with ADHD, which also affect the way they interact with peers and respond to social situations [43]. In middle school students, emotional self-awareness and emotional control mediated the relationship between ADHD symptoms and poor social skills [43]. The aforementioned study was replicated by Cleminshaw and colleagues using an adolescent ADHD population where emotional dysregulation and presence of ADHD mediated parent-rated social skills of adolescents with ADHD [44]. Overall, difficulties with one or more of the above factors may be contributing to the both the reduced number of friends and poorer friendship stability, quality and interactions for children and youth with ADHD.

### Objectives and research questions

Therefore, the aim of this systematic review was to systematically review and methodologically appraise the existing evidence of studies reporting on friendships of children and youth with ADHD. Our conceptualization of friendship included four domains: *presence of friendship*, *friendship quality*, *characteristics of friends* and *friendship interactions*. This study was guided by the following research questions:

1. What is the study design and reported aspects of friendship for children and youth with ADHD?
2. How do the friendships of children and youth with ADHD compare to TD children?
3. What associations are reported between friendship and social-emotional wellbeing and mental health in children and youth with ADHD?
4. What is the methodological quality of studies reporting on the friendships of children and youth with ADHD?

## Methods

A Measurement Tool to Assess Systematic Reviews informed the methodological design of this systematic review [46]. Additionally, the Preferred Reporting Items for Systematic Reviews and Meta-Analysis (PRISMA) statement and checklist guided transparent reporting of this systematic review in 27-item areas from the title to the discussion [47].

### Protocol and registration

As per the PRISMA statement, the protocol was registered with the International Prospective Register of Systematic Reviews (PROSPERO registration ID CRD42021213718). The protocol may be accessed via the following URL: https://www.crd.york.ac.uk/prospero/display_record.php?ID=CRD42021213718.

### Eligibility criteria

Eligibility criteria were developed prior to the database search. Included studies were required to meet the following criteria: (1) participants needed to be aged 18 years or younger; (2) participants required a confirmed ADHD diagnosis by a qualified professional (e.g. psychiatrist or paediatrician) using recognised diagnostic tools such as the Diagnostic and Statistical Manual (DSM) of Mental Disorders 4^th^ or 5^th^ edition (DSM-III-R [48]; DSM-IV; [49] DSM-IV-TR; [50]; DSM-5; [1]), World Health Organisation (WHO) International Classification of Diseases 10^th^ Edition, (ICD-10; [51]) or Diagnostic Interview Schedule for Children Version IV (DISC-IV; [52]); (3) participants could have multiple diagnoses (e.g. ADHD and ODD) provided ADHD was the primary diagnosis; (4) measurement of friendship needed to be related to at least one of the adopted friendship domains (i.e., *presence of friendship*, *friendship quality*, *characteristics of their friends*, and *friendship interactions*); and (5) measurement could take the form of peer nominations, self- or parent-report friendship questionnaires, behavioural reports from parents and teachers, or clinician observations. Studies that used a quantitative study design, including exploratory designs, were eligible for inclusion.

Studies were excluded if they: (1) had participants described ‘at risk’ or used only parent and teacher reports of ADHD symptomology; (2) had participants where ADHD was a secondary condition (e.g., in addition to autism spectrum disorder); (3) reported on other aspects of social skills or peer functioning such as peer status, or peer problems without reference to friendship; (4) reported on outcomes after friendship intervention; and (5) were not published in a peer-reviewed journal or, were published in a language other than English.

### Information sources and search strategy

The database selection and search strategy were developed in consultation with the first and third authors in collaboration with the Academic Liaison Librarian at the University of Sydney. A comprehensive search was completed across six databases: CINAHL, Eric, Embase, MEDLINE, PsycInfo and SCOPUS (see Table 1). The first author conducted the search on March 3, 2021, with no date limit applied. By identifying key words from similar reviews on friendship, this shaped the search strategy into three elements: (1) search terms related to ADHD; (2) search terms related to friendship and; (3) search terms related to both children and adolescence (see Table 1). A grey literature search using internet sources (Google Scholar) and manually searching reference lists was conducted by the first author to identify any additional studies.

**Table 1:** Search strategy implemented across databases.

Database	Search terms	Limitations
**CINAHL**	[(MH “Attention Deficit Hyperactivity Disorder”) OR “attention deficit hyperactivity disorder” OR “attention deficit disorder” OR “hyperactive” OR (MH “Impulsive+”) OR “impulsive*” OR “inattenti*” OR “ADHD”] AND [(MH “Friendship”) OR “friend*” OR “acquaintance” OR “companion” OR “peer relation*”] AND [(MH “Child+”) OR “child*” OR (MH “Adolescence+”) OR”adolescen*” OR “teen”]	Language: English
**EMBASE**	(attention deficit hyperactivity disorder.mp. or attention deficit disorder/ OR ADHD.mp. OR hyperactivity/ or hyperactiv*.mp. OR impulsiveness/ or impulsiv*.mp. OR inattenti*.mp.) AND (friend/ or friend*.mp. OR acquaintance.mp. OR companion*.mp. OR peer relation*.mp) AND (child*.mp. or child/ OR adolescent/ or adolescence/ or adolescen*.mp. OR teen*.mp.)	Language: English
**ERIC**	(attention deficit hyperactivity disorder.mp. or exp Attention Deficit Hyperactivity Disorder/ or exp Attention Deficit Disorders/ OR ADHD.mp. OR hyperactiv*.mp. OR exp Hyperactivity/ OR impulsiv*.mp. OR inattenti*.mp.) AND (exp Friendship/ or friend*.mp. OR acquaintance.mp. OR companion*.mp. OR exp Peer Relationship/ or peer relation*.mp.) AND (child*.mp. OR children.mp. or exp Children/ OR teen*.mp. OR exp Adolescents/ or adolescen*.mp.)	Language: English
**Medline**	(attention deficit hyperactivity disorder.mp. or Attention Deficit Disorder with Hyperactivity OR ADHD.mp. OR hyperactiv*.mp. OR Impulsive Behavior/ or impulsiv*.mp. OR inattenti*.mp.) AND (Friends/ or friend*.mp. OR acquaintance.mp. OR companion*.mp. OR peer relation*.mp.) AND (child*.mp. or Child/ OR teen*.mp. OR Adolescent/ or adolescen*.mp.)	Language: English
**PsycINFO**	(ADHD.mp. or exp Attention Deficit Disorder with Hyperactivity OR attention deficit disorder.mp. or exp Attention Deficit Disorder OR attention deficit hyperactivity disorder.mp. OR hyperactiv*.mp. OR impulsiv*.mp. OR inattenti*.mp.) AND (exp Friendship/ or friend*.mp. OR acquaintance.mp. OR companion*.mp. OR exp Peer Relations/ or peer relation*.mp.) AND (child*.mp. OR teen*.mp. OR adolescen*.mp.)	Language: English
**SCOPUS**	((“ADHD” OR “Attention deficit hyperactivity disorder” OR “attention deficit disorder” OR “hyperactive*” OR “Inattenti*” OR “Impulsiv*”)) AND ((“friend*” OR “peer relation*” OR “companion*” OR “acquaintance”)) AND ((child*” OR “teen*” OR “adolescen*”))	Language: English

### Study selection

Study selection was performed by the first and seventh authors on the title and abstract of records. Prior to this, inter-rater agreement was established through a training session led by the first author on the eligibility criteria whereby both authors screened the same 10 title and abstracts independently using the eligibility criteria. A 100% inter-rater agreement was achieved. Thereafter, the first author independently screened all title and abstracts against the eligibility criteria and then, to ensure rating accuracy, 50% of the title and abstracts were randomly selected and screened by the seventh author. The Covidence software (https://www.covidence.org) was used for screening as it is the primary screening and data extraction tool recommended by the Cochrane Community [53]. Authors scored records as *yes* or *no* based on the eligibility criteria. Disagreements were discussed with the second and fifth authors to reach consensus. Weighted Kappa with linear weights was calculated to assess inter-rater agreement.

### Data collection and extraction

In alignment with the Cochrane Handbook for Systematic Reviews of Interventions, two data extraction tables were created, trialled on studies, and refined based on feedback from the first, second, third and fifth authors [54]. One table pertained to participant and study characteristics, including: study design, country of study, sources of participants, participant age, gender and comorbidities, eligibility criteria, friendship outcome and measures, source(s) of data, and methodological quality. The second table included: the friendship findings of each study mapped against our conceptualisation of friendship, the associations between friendship and social-emotional wellbeing and mental health (if reported), and the main findings reported in the included studies, with a focus on comparing friendships to TD children. The first author extracted all data. Authors from one of the included studies were contacted via email for a subset of data as the age range of the sample exceeded 18 years old, but the mean was under 18 years [55].

#### Mapping against our conceptualization of friendship

Findings about friendships reported in the included studies were mapped under four friendship domains adopted for the study, based on Hartup’s [16] and Bagwell and Schmidt’s [21] friendship models. These were: *presence of friendship* (i.e., whether a child has a friend, the number of their reciprocated friendships and the stability of their friendships), *friendship quality* (i.e., positive and negative features such as companionship, support or conflict), *characteristics of friends* (i.e., attitudes, interests, and social behaviours or competencies of friends), and *friendship interactions* (i.e., frequency of contact with friends, the quality of friendship interactions and self-perception of competence). Authors 1,2, 3 and 5 engaged in discussion to determine the domains the extracted measurements of friendship mapped against.

#### The role and impact of friendship

The factors associated with friendship were extracted when studies conducted secondary analyses that investigated friendship as a correlation, mediating, or moderating factor on children’s social-emotional wellbeing and mental health. Studies were considered to investigate the associations with friendship if friendship data was included in correlation statistical analyses and impact if friendship data was used in mediating or moderating statistical analyses.

### Methodological quality

To assess the methodological quality of included studies, the Standard Quality Assessment Criteria for Evaluating Primary Research Papers from a Variety of Fields (QualSyst checklist) was administered as it can be used for all quantitative studies [56]. The Appraisal tool for Cross-Sectional Studies (AXIS) was also applied, as most included studies used a cross-sectional design [57].

The first author independently rated studies against the QualSyst checklist and AXIS. The seventh author also rated a randomly selected 50% overlap on the included studies using the QualSyst checklist. If disagreements arose, consensus was reached through discussion with the first, second and seventh authors. The fourth author also rated a randomly selected 50% overlap on the included studies using the AXIS checklist. Weighted kappa with linear weights was calculated to determine inter-rater agreement. To achieve a quality score using the QualSyst checklist, fourteen items were rated as yes = 2, partial = 1, no = 0 or not applicable to achieve a maximum score of 28, which is reported as a percentage. Items marked not applicable were excluded from the calculation. Studies were scored as strong (>80%), good (60–79%), adequate (50–59%) or poor quality (<50%) [56]. The AXIS has 20 items which are rated yes, no or don’t know/comment and no quality score is calculated [57]. The proportion of the 20 items adequately addressed (i.e., scored yes) was calculated.

## Results

### Study selection

**Fig 2:**
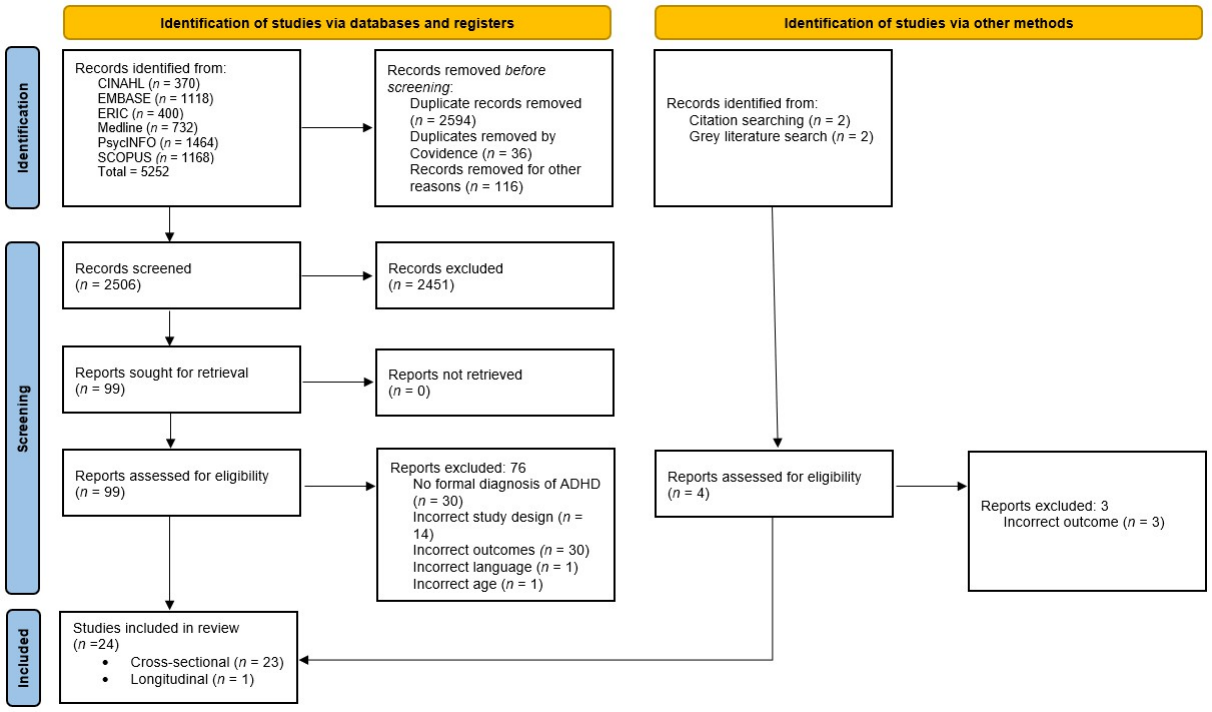
PRISMA flow diagram.

In total, 5252 abstracts were retrieved from six databases. Duplicate abstracts (n = 2630) and other publication types (i.e., conference abstracts, reports; n = 116) were excluded prior to screening, leaving 2506 records to be imported into Covidence (https://www.covidence.org) for title and abstract screening. The first author independently screened all 2506 title and abstracts with 1253 (50%) randomly selected records screened independently by the seventh author. There was strong inter-rater agreement kw = 0.81 (95% CI = 0.76–0.86). 99 studies were identified for full text review, with 23 of the 99 studies meeting the inclusion criteria along with one additional study [58] identified during handsearching. Due to the broad search strategy, a significant number of studies were excluded as irrelevant abstracts such as pharmacological and neurological imaging abstracts were captured in the database search. A total of twenty-four studies were included for data extraction. Fig 2 shows a PRISMA flow diagram detailing the study selection process.

### Study characteristics

Of the twenty-four studies, twenty-three studies were cross-sectional and one was longitudinal [24]. Most studies were conducted in Canada (*n* = 11), with the remaining conducted in the United States, Australia, Bahrain, China, Denmark, Greece and Israel (see Table 2).

**Table 2:** Summary of participant and study characteristics.

Author; Year; Country	Study design; Participant sources	Participants: Mean age (*SD*) years; Comorbidities; Male% ADHD/TD	Eligibility criteria (Diagnosis method; Rating tool; IQ; Exclusion)	Friendship outcomes; Measures of friendship	Source of friendship data	Quality (QualSyst^a^; AXIS^b^)
Al Ansari, Hamadeh [55]; Bahrain	Cross-sectional; CAPU	*ADHD*: *n* = 23 *Age*: 15.87 (1.14) *Comorbidities*: NR Male%: 74	*Diagnosis*: DSM-IV *Tool*: CRPR/CTRS-R: L *IQ*: > 80 *Exclusion*: ASD	*Outcomes*: Quantity *Measures*: Telephone questionnaire, “Has your child got any close friends?”	*Self*: *Parent*: X *Observation*:	*QualSyst*: Good (75%) *AXIS*:14/20
Al-Yagon [59]; Israel	Cross-sectional; Schools	*ADHD-LD*: *n* = 91 *Age*:15.94 (0.7) *Comorbidities*: LD *TD*: *n* = 99 *Age*: 15.94 (0.7) Male%: 45 / 45	*Diagnosis*: DSM-IV-TR by psycho-education team *Tool*: Conners-3 parent, teacher and self *IQ*: NR *Exclusion*: NR	*Outcomes*: Quality *Measures*: FQQ	*Self*: X *Parent*: *Observation*:	*QualSyst*: Strong (90%) *AXIS*: 15/20
Bagwell, Molina [10]; United States	Cross-sectional; Summer Treatment Program, community, schools	*ADHD*: *n* = 111 *Age*: 15.09 (1.47) *Comorbidities*: CD *TD*: *n* = 100 *Age*: 15.09 (1.47) Male%: 96	*Diagnosis*: DSM -III-R or DSM-IV *Tool*: *DBD*, *parent and teacher report* *IQ*: > 80 *Exclusion*: Seizures, psychosis, sexual other neurological dx PDD,	*Outcomes*: self-perception, quantity friend characteristics *Measures*: Harter Self-Perception Scale for Adolescents [60], one item on the CBCL (no. of close friends), Conventional Activities of Friends Scale [61], substance use measure adapted Monitoring the Future study [62]	*Self*: X *Parent*: *X* *Observation*:	*QualSyst*: Strong (83%) *AXIS*: 12/20
Blachman and Hinshaw [30]: United States	Cross-Sectional; Medical settings, mental health settings, paediatric settings, schools, community	*ADHD-Combined*: *n* = 93 *Age*: 9.5 (1.7) *ADHD-Inattentive*: *n* = 47 *Age*: 9.8 (1.7) *Comorbidities*: Anxiety, depression, disruptive behaviour disorders *TD*: *n* = 88 *Age*: 9.4 (1.65) Male%: 0/0	*Diagnosis*: WISC-III and DISC-IV by highly trained graduate student *Tool*: DISC-IV, SNAP-IV parents ratings *IQ*: > 70 *Exclusion*: Neurological damage, psychosis, PDD	*Outcomes*: Quantity, Stability/Duration, Quality *Measures*: Sociometric measures, FQM	*Self*: X *Parent*: *Observation*:	*QualSyst*: Strong (89%) *AXIS*: 14/20
Cardoos and Hinshaw [63]; United States	As above Blachman and Hinshaw [30]	As above Blachman and Hinshaw [30]	As above Blachman and Hinshaw [30]	*Outcomes*: Quantity *Measures*: Sociometric measures	*Self*: X *Parent*: *Observation*:	*QualSyst*: Strong (89%) *AXIS*: 14/20
Elmose and Lasgaard [64]; Denmark	Cross-sectional; Two special education schools	*ADHD*: *n* = 25 *Age*: 14.6 (1.04) *Comorbidities*: All *TD*: *n* = 199 *Age*: 14.1 (0.43) Male%: 100 /100	*Diagnosis*: ICD-10 criteria for F90 Hyperkinetic disorder from CAPU *Tool*: None *IQ*: > 70 *Exclusion*: ASD, ID	*Outcomes*: PSS, Difficulty Making Friends *Measures*: SSSC, 4-point scale to measure difficulty making friends; strongly disagree (1) to strongly agree (4)	*Self*: X *Parent*: *Observation*:	*QualSyst*: Strong (85%) *AXIS*:15/20
Heiman [33]; Israel	Cross-sectional; Schools	*ADHD*: *n* = 39 *Age*: 11.2 (2.05) *Comorbidities*: *TD*: *n* = 17 *Age*: 10.1 (1.1) Male%: 79/70	*Diagnosis*: DSM-IV and WISC-III by psychologist *Tool*: CPRS/CTRS *IQ*: 88–120 *Exclusion*: ASD, ID	*Outcomes*: Quantity, contact with friends *Measures*: Friendship Quality Questionnaire Children and Adult version [65]	*Self*: X *Parent*: X *Observation*:	*QualSyst*: Good (78%) *AXIS*: 12/20
Houghton, Lawrence [66]; Australia	Cross-sectional; Schools, community	*ADHD*: *n* = 42 *Age*: 13.4 (2) *Comorbidities*: NR *Non-ADHD*: *n* = 42 *Age*: 13.4 (2) Male%: 74 / 74	*Diagnosis*: DSM-IV-TR or DSM-5 from paediatrician or child psychiatrist. *Tool*: None *IQ*: NR *Exclusion*: NR	*Outcomes*: Quality *Measures*: Perth Aloneness Scale	*Self*: X *Parent*: *Observation*:	*QualSyst*: Strong (95%) *AXIS*: 16/20
Hoza, Mrug [31]; United States	Cross-sectional; Mental health settings, schools, community, paediatricians	*ADHD*: *n* = 165 *Age*: 7.7 (0.8) *Comorbidities*: CD, ODD, Anxiety, specific learning disabilities *TD*: *n* = 165 *Age*: 7.7 (0.8) Male%: 79 / 79	*Diagnosis*: DSM-IV *Tool*: DISC- IV (parent and teacher report) *IQ*: > 80 *Exclusion*: psychosis, neurological, bipolar, personality disorders, medically unwell in hospital, family member in study, Tourette’s syndrome.	*Outcomes*: Quantity *Measures*: Sociometric nominations	*Self*: X *Parent*: *Observation*:	*QualSyst*: Strong (83%) *AXIS*: 17/20
Jia and Mikami [67]; United States	Cross-sectional; Schools, community, paediatricians	*ADHD*: *n* = 24 *Age*: 8.15 (0.79) *Comorbidities*: ODD, internalising *TD*: *n* = 133 *Age*: 8.19 (0.83) Male%: 54 / 40	*Diagnosis*: KSADS *Tool*: CSI and Teacher-Peer Social Skills Questionnaire *IQ*: > 80 on WASI *Exclusion*: ASD	*Outcomes*: Quantity *Measures*: Sociometric nominations	*Self*: X *Parent*: *Observation*:	*QualSyst*: Strong (90%) *AXIS*: 14/20
Kouvava and Antonopoulou [32]; Greece	Cross-sectional; Schools	*ADHD*: *n* = 40 *Age*: 9.75 (1.21) *Comorbidities*: None *TD*: *n* = 120 *Age*: 9.43 (1.06) Male%: 53 / 44	*Diagnosis*: DSM-5 *Tools*: None *IQ*: NR *Exclusion*: Only children and twins, no comorbidities	*Outcomes*: Quantity, quality, characteristics of friends, stability *Measures*: FQQ, sociometric nominations, friendship interview from parents and children	*Self*: X *Parent*: X *Observation*:	*QualSyst*: Strong: (85%) *AXIS*: 14/20
Ma, Lai [68]; China	Cross-sectional; Psychiatry clinic	*ADHD*: *n* = 113 *Age*: 8.12; NR Male%: 79	*Diagnosis*: DSM-5 *Rating tools*: NR *IQ*: NR *Exclusion*: NR	*Outcomes*: PSS *Measures*: SSSS	*Self*: X *Parent*: *Observation*:	*QualSyst*: Strong (90%) *AXIS*: 17/20
Marton, Wiener [40]; Canada	Cross-sectional; Hospitals, community	*ADHD*: *n* = 50 *Age*: 10.08 (1.39) *Comorbidities*: LD, anxiety, ODD, mood disorder *TD*: *n* = 42 *Age*: 10.20 (1.46) Male%: 72 / 71	*Diagnosis*: DSM-IV from a physician or mental health professional. *Tool*: CPRS/CTRS-R:L *IQ*: > 80 *Exclusion*: ASD	*Outcomes*: Quantity, stability, characteristics of friends, frequency of contact *Measures*: Friendship interview and questionnaire	*Self*: X *Parent*: X *Observation*:	*QualSyst*: Strong (95%) *AXIS*: 16/20
Mastoras, Saklofske [69]; Canada	Cross-sectional; Schools, community, psychology clinics	*ADHD-C/HI*: *n* = 55 *Age*: 9.99 (1.16) *Comorbidities*: LD, anxiety, ODD, DCD Male%: 87	*Diagnosis*: Psychologist or physician *Tool*: Conners-3 parent *IQ*: > 85 on WASI *Exclusion*: ADHD-Inattentive, ASD, psychosis, other neurological dx	*Outcomes*: PSS *Measures*: CASS	*Self*: X *Parent*: *Observation*:	*QualSyst*: Strong (95%) *AXIS*: 16/20
Maya Beristain and Wiener [70]; Canada	Cross-sectional; Community mental health services	*ADHD*: *n* = 59 *Age*: 15.19 (1.48) *Comorbidities*: LD, ODD, CD, anxiety, depression *TD*: 48 *Age*: 15.23 (1.64) Male%: 64 / 48	*Diagnosis*: From psychologist or physician *Tool*: Conners-3 parent, teacher, self-report *IQ*: >85 on WASI *Exclusion*: NR	*Outcomes*: Quantity, stability, characteristics of friends, frequency of contact *Measures*: AFQ, PFQ	*Self*: X *Parent*: X *Observation*	*QualSyst*: Strong (95%) *AXIS*: 16/20
Normand, Schneider [27]; Canada	Cross-sectional; Paediatric clinics, community schools	ADHD: *n* = 87 *Age*:10.30 (1.85) *Comorbidities*: Anxiety and ODD *TD*: *n* = 46 Male%: 77 / 74 *Age*:10.41 (1.72) *Friend (ADHD)*: *n* = 87 *Age*:10.39 (2.22) *Friend (TD)*: *n* = 46 *Age*: 10.22 (1.68) Male%: 69 / 70	*Diagnosis*: Psychologists, paediatricians, psychiatrists and/or family physicians *Tool*: CPRS/CTRS-R: L *IQ*: > 80 *Exclusion*: PDD, psychosis, no friend to participate or not in a regular classroom	*Outcomes*: Quantity, quality, quality of interactions, characteristics of friends, stability *Measures*: Friendship nominations, FQM, Car-Race Task, card sharing and game-choice task	*Self*: X *Parent*: *Observation*: X	*QualSyst*: Strong (90%) *AXIS*: 14/20
Normand, Schneider [24]; Canada	Longitudinal study; 6-month follow-up of Normand et al., 2011	*ADHD*: *n* = 71 *Age*:10.22 (1.92); *Comorbidities*: LD, ODD, anxiety, DCD, attachment disorder *TD*: n = 44 *Age*:10.22 (1.92) Male%: NR	As above in Normand et al. (2011)	As above in Normand et al. (2011) except for characteristics of friends.	As above in Normand et al. (2011)	*QualSyst*: Strong (95%) *AXIS*: N/A
Normand, Ambrosoli [71]; Canada	As above in Normand et al., 2011	As above in Normand et al., 2011	As above in Normand et al., 2011	*Outcomes*: Quantity, quality of interactions *Measures*: Friendship nominations, Car-Race Task and free-play task	*Self*: X *Parent*: *Observation*: X	*QualSyst*: Strong (80%) *AXIS*: 11/20
Normand, Soucisse [72]; Canada	As above in Normand et al., 2011	As above in Normand et al., 2011	As above in Normand et al., 2011	*Outcomes*: Quantity, quality of interactions *Measures*: Friendship nominations, free-play task	*Self*: X *Parent*: *Observation*: X	*QualSyst*: Strong (95%) *AXIS*: 14/20
Normand, Mikami [34]; Canada	Cross-sectional; Hospitals, clinics, schools	*ADHD*: *n* = 165 *Age*: 8.59 (1.51) *Comorbidities*: externalising, internalising Male%: 67	*Diagnosis*: DSM-5 *Tool*: CSI-IV and SDQ *IQ*: > 75 *Exclusion*: ASD, other dx	*Outcomes*: Quality *Measures*: FQQ-SF	*Self*: X *Parent*: X *Observation*:	*QualSyst*: Strong: (90%) *AXIS*: 16/20
Redmond [73]; United States	Cross-sectional; Community, psychologists	*ADHD*: *n* = 20 *Age*: 7.86 (0.62) *Comorbidities*: None TD: *n* = 20 *Age*: 7.83 (0.53) Male%: 75 / 55	*Diagnosis*: Healthcare professional *Tool*: CBCL DSM-ADHD *IQ*: NR *Exclusion*: ASD, PDD or LI	*Outcome*: Quantity, frequency of contact *Measures*: Two items from CBCL social competence sub-scale	*Self*: *Parent*: X *Observation*:	*QualSyst*: Strong (90%) *AXIS*: 13/20
Rokeach and Wiener [74]; Canada	Cross-sectional; Mental health services, community, previous participants	*ADHD*: *n* = 61 *Age*: 15.28 (1.54) *Comorbidities*: LD, ODD, CD, anxiety, mood disorders *TD*: *n* = 54 *Age*: 15.41 (1.75) Male%: 66 / 46	*Diagnosis*: DSM-IV from a physician or mental health professional *Tool*: Conners-3 parent, teacher, self-report *IQ*: > 80 on WASI *Exclusion*: As above in IQ	*Outcomes*: Quality *Measures*: Networks of Relationships Inventory: Behavioural Systems Version	*Self*: X *Parent*: *Observation*:	*QualSyst*: Strong: (95%) *AXIS*: 15/20
Smit, Mikami [75]; Canada	Cross-sectional; Schools, hospitals, clinics	*ADHD*: *n* = 213 *Age*: 8.58 (1.55) *Comorbidities*: Externalising, internalising Male%: 69	*Diagnosis*: K-SADS/DSM-5 *Tool*: CSI-IV and SDQ *IQ*: >75 on WASI *Exclusion*: ASD, other dx	*Outcomes*: Quantity, quality of interactions *Measures*: Sociometric nominations, observational toy sharing task and car-race game	*Self*: X *Parent*: *Observation*: X	*QualSyst*: Strong (95%) *AXIS*: 16/20
Timmermanis and Wiener [58]; Canada	Cross-sectional; Practitioner, community, previous participants	*ADHD*: *n* = 40 *Age*: 14.65 (1.70) *Comorbidities*: LD, CD, anxiety, depression, ODD, *TD*: *n* = 24 *Age*: 15.14 (1.42) Male%: 68 / 54	*Diagnosis*: DSM-IV, *Tool*: Conners-3 parent, teacher, self-report *IQ*: > 80 on the WASI *Exclusion*: PDD, ID, OCD psychotic, bipolar, Tourette’s Disorder	*Outcomes*: PSS *Measures*: SS-B	*Self*: X *Parent*: *Observation*:	*QualSyst*: Strong (90%) *AXIS*: 15/20

*Notes*. ADHD = Attention-Deficit Hyperactivity Disorder; AFQ = Adolescent Friendship Questionnaire [76]; ASD = Autism Spectrum Disorder; AXIS = Appraisal tool for Cross-Sectional Studies [57]; CAPU = Child and Adolescent Psychiatry Unit; CASS = Child and Adolescent Social Support Scale [77]; CBCL = Child Behaviour Checklist [78]; CPRS/CTRS-R: L = Conners Parent Rating Scale/Conners Teachers Rating Scale [79, 80]; Conners-3 [80]; CSI = Child Symptom Inventory [81]; CSI-IV = Child Symptom Inventory-4 [81]; DBD = Disruptive Behaviours Disorders rating scale [82]; DCD = Developmental Coordination Disorder; DISC-IV = Diagnostic Interview Schedule for Children Version IV [52]; DSM-5 = Diagnostic and Statistical Manual Disorders 5th edition [1]; DSM-IV = Diagnostic and Statical Manual of Mental Disorders 4th edition [49]; DSM-IV-TR = Diagnostic and Statical Manual of Mental Disorders 4th edition, text revised [50]: Dx = Diagnosis; Friendship interview and questionnaire [22]; FQM = Friendship Qualities Measure [83]; FQQ = Friendship Quality Questionnaire [25]; FQQ-SF = Friendship Quality Questionnaire—Short Form [84]; ID = Intellectual disability; IQ: Intelligence Quotient; KSADS = Kiddie Schedule for Affective Disorders and Schizophrenia [85]; LD = Learning disability; LI = Language Impairment; Networks of Relationships Inventory–Behavioural Systems Version [23]; NR = not reported; Observational Car-Race Task [86]; OCD = Obsessive compulsive disorder; PFQ = Parent Friendship Questionnaire [76]; Perth Aloneness Scale [87]; PDD = Pervasive developmental disorder; PSS = Perceived Social Support; SDQ = Strengths and Difficulties Questionnaire Peer Problems subscale [88]; SNAP = Swanson, Nolan and Pelham Rating Scale [89] SS-B = Social Support Behaviors Scale [90]; SSSC = Social Support Scale for Children [91]; SSSS = Student Social Support Scale [92, 93]; TD = Typically Developing participants; Teacher-Peer Social Skills Questionnaire [94]; WASI = Wechsler Abbreviated Scale of Intelligence [95, 96]; WISC-III = Weschler Intelligence Scale for Children [97]

^a^QualSyst Rating reported as Strong, Good, Adequate or Poor with percentage score

^b^AXIS score = number of Yes votes / total number of criteria (*n* = 20)

### Participants

Across the twenty-four studies, there were 1509 participants with ADHD with a mean age of 11.4 years (range 5–18 years; *SD* = 3.0). A total of 1197 TD participants with a mean age of 11.8 years (range 6.8–18 years; *SD* = 3.1) were included across nineteen studies that used a TD comparison group. Participants with ADHD were mostly male, 68.4% and 63.7% of TD participants were male. The IQ of participants varied between 70–120 on the Wechsler Abbreviated Scale of Intelligence [95, 96], though five studies did not report IQ eligibility criteria [59, 66, 68, 73]. In five studies, participants’ diagnosis was confirmed using the DSM-5, seven studies used the DSM-IV, two studies used the DSM-IV-TR, DISC-IV or the KSADS [85], and one study used the ICD-10 F90 Hyperkinetic disorder criteria [51] or DSM-III-R [51]. Seven studies did not report the diagnosis confirmation tool (see Table 2). Twenty studies used rating scales to confirm ADHD symptomology, with the Conners rating scale-revised or Conners-3 [79, 80] most commonly used across twelve studies (see Table 2).

### Friendship outcomes

Regarding the first research question, nine primary friendship outcomes were identified across the twenty four included studies: friendship quantity (*n* = 16), friendship stability *(n* = 6), difficulty making friends (*n* = 1), perceived social support (PSS) from friends (*n* = 4), friendship quality (*n* = 9), self-perception of competence in friendship (*n* = 1), the quality of interactions with friends (*n* = 5), frequency of contact with friends (*n* = 4) and characteristics of friends (*n* = 5; see Table 2). These reported outcomes were then mapped against our four friendship domains (see Table 3). The most investigated domain was the *presence of friendships* (*n* = 17), which included reported outcomes of friendship quantity, friendship stability and difficulty making friends. This was followed by the second domain, *friendship quality* (*n* = 12), which included reported outcomes on friendship quality and PSS from friends. The third domain, *friendship interactions* (*n* = 10), included the reported outcomes of quality of interactions, perception of friendship competence and frequency of contact. The fourth domain, c*haracteristics of friends* (*n* = 5) included reported outcomes on the characteristics of friends (see Table 3).

**Table 3:** Summary of friendship outcomes.

Study	Friendship Domain Outcomes^a^					Main findings
	Presence of friends	Friendship Quality	Characteristics of friends	Friendship Interactions	Role/ Impact^b^	
Al Ansari, Hamadeh [55]						35% of youth reported having no close same-sex friends.
Al-Yagon [59]						No significant difference in youth friendship quality of ADHD-LD and TD Youth with ADHD-LD with higher quality friendships reported significantly more positive affect (*p* < .01) and significantly less peer-network/dyadic loneliness (*p* < .01) than those with lower quality friendships Higher quality friendships showed a non-significant reduction in negative affect
Bagwell, Molina [10]						Parents of adolescents with childhood ADHD reported significantly fewer close friends than the comparison group (*p* < .001) and were reported to be involved in less conventional activities that non-ADHD adolescents (*p* < .01) No significant difference reported between adolescents with childhood ADHD and without ADHD in their self-perception of friendship competence and substance use Adolescents with persistent ADHD had significantly fewer parent-reported friends (*p* < .05) and friends who engaged in fewer conventional activities (*p* < .05) compared to non-ADHD peers. Parents of adolescents with childhood ADHD thought their friends were a bad influence regardless of adolescent ADHD status (*p* < .05) Adolescents with persistent ADHD/CD reported more of their friends engaged with substances (*p* < .05) and significantly less involved in conventional activities (*p* < .01)
Blachman and Hinshaw [30]						Girls with ADHD (both combined and inattentive) reported fewer friends compared to TD girls (*p* < .001). Girls with ADHD (both groups) were more likely to have no friends compared to TD peers (*p* < .01) Girls with ADHD-Combined type had more difficulty initially maintaining any stable friendships from the start to middle of program (*p* < .005) whereas girls with ADHD-Inattentive type had more difficulty keeping more than one consistent friendship in the latter part of the program (*p* < .005) Girls with ADHD-Combined type and ADHD-Inattentive type had significantly higher ratings of negative friendship quality compared to TD peers (*p* < .001)
Cardoos and Hinshaw [63]						Girls with ADHD had significantly fewer friends than comparison girls (*p* = .026). In girls with ADHD, 49 girls reported no-friends and 89 had at least one friend whereas, in the comparison group, only 19 girls had no friends and 69 had at least one friend. ADHD Girls with internalising or externalising behaviours with no reported friends were significantly more likely to experience peer victimisation (*p* < .001) than girls with at least one friend. The presence of at least one mutual friendship moderated the association between behavioural risk (internalising, externalising behaviours and social competence) and victimisation, such that the presence of a friend reduced victimisation (*p* < .05)
Elmose and Lasgaard [64]						Boys with ADHD had more difficulty making friends than TD youth (*p* < .01) PSS from close friends significantly reduced loneliness (*p* < .01) No significant differences in loneliness between TD boys and boys with ADHD No significant association between loneliness and difficulty making friends
Heiman [33]						Children with ADHD were significantly more likely to meet friends at school and in the playground, whereas TD peers more likely at home (*p* < .001) No significant differences reported on how children with and without ADHD make friends Parents and teachers reported a higher number of friends for TD peers compared to ADHD children (*p* < .01)
Houghton, Lawrence [66]						Children with ADHD reported significantly lower friendship quality (*p* = .005) Friendship quality significantly reduced no. of depressive symptoms (*p* < .001) Friendship quality and feelings of isolation (having few friends or lack of PSS) mediated the relationship between ADHD and depressive symptoms (*p* < .001)
Hoza, Mrug [31]						Children with ADHD had significantly less friendships than TD peers (*p* < .001) For children with ADHD, 56% reported no dyadic friendships, 33% had one dyadic friendship and 9% had two dyadic friendships For TD children, 32% had no dyadic friends, 39% had one dyadic friend and 22% had two dyadic friends Children with comorbid anxiety had fewer dyadic friends (*p* < .05)
Jia and Mikami [67]						Children with ADHD reported fewer friends than TD peers (*p* = .027) Friendship quantity moderated externalising behaviour and bully status; more friends exacerbated bully status (*p* = .002); more positive for boys (*p* < .001) Boys with ADHD-externalising behaviour, increased no. of friends was protective function from victimisation (*p* = .020) Girls with ADHD-externalising behaviour, increased no. of friends was less protective function from victimisation (*p* = .003)
Kouvava and Antonopoulou [32]						Children with ADHD had significantly fewer mutual friends (*p* = .000) fewer stable friendships (*p* = .000), less positive features and more conflict compared to TD peers (*p* = .000) and more likely to have friends with learning disability (*p* = .000) On average children with ADHD had one friend; TD children had two friends Quality of sibling relations significantly predicted no. of mutual friendships for children with ADHD (*p* < .001)
Ma, Lai [68]						Children with ADHD reported PSS from friends least available Children with ADHD ranked PSS from friends as third most important out of four sources (mother, father, teacher) Increased PSS from friends significantly increased overall perceived sense of competences and hope (*p* < .01) PSS from friends significant for children’s sense of cognitive competence, (*p* < .01) and overall hope (*p* < .05) and agency (*p* < .01)
Marton, Wiener [40]						No significant between-group difference in no. of nominated friends for children (3 friends ADHD; 4 friends TD). Significantly less friends corroborated by parents and/or teacher of children with ADHD (*p* = .008) Children with ADHD had significantly shorter friendships than TD (*p* < .05) and a higher proportion of their friends had learning/behaviour problems (*p* = .000) Non-significant but less contact with friends outside of school compared to TD peers (Child- and parent-report) Children with ADHD reported having 9–14 months friendships shorter than TD
Mastoras, Saklofske [69]						Children with ADHD reported lower PSS from friends (*p* < .03) Increased PSS from friends significantly increased self-worth (*p* < .01) Increased PSS from friends not associated with decreased depressive/anxiety symptoms or increased self-reliance, school competence or social acceptance
Maya Beristain and Wiener [70]						No significant differences in the no. of nominated friends, friendship duration and frequency of contact between youth with and without ADHD Fewer corroborated friends for females with ADHD than males with ADHD; fewer corroborated friends for TD males than TD females (*p* < .05) Youth with ADHD more likely to have a two-year age gap with friends (*p* = .02) Less likely for females with ADHD to go to school with best friend (*p* = .03) Youth with ADHD more likely to have friends with behaviour problems (*p* = .01)
Normand, Schneider [27]						Average reported friendship length for children with ADHD 4.33 years (*SD*: 2.99); Average for comparison children 4.8 years (*SD*: 3.12) Children with ADHD reported poorer friendship quality than TD peers (*p* < .05) Invited friends reported less positive features compared to invited friends of TD children (*p* < .001). Less friendship satisfaction in ADHD dyads (*p* < .01) Children with ADHD had more friends displaying ADHD symptoms Children with ADHD made significantly more legal and illegal moves *(p* < .01*)*, insensitive (*p* < .01), self-centred (*p* < .001) proposals No significant differences for ADHD/ODD and ADHD children. Children with ADHD/anxiety less self-centred proposals (*p* < .01) and illegal moves (*p* < .05)
Normand, Schneider [24]						After six months (Time 2) 75% of children with ADHD had the same friend compared to 91% TD children (*p* = .02) At Time 2, children with ADHD perceived poorer friendship quality (*p* < .05) Invited friends perceived fewer positive features (*p* < .001) and more negative features (*p* < .01). ADHD dyads had less friendship satisfaction (*p* < .05) Illegal moves, self-centred and insensitive proposals reduced in TD children (*p <* .*001*) and increases in children with ADHD (*p* < .001) Insensitive negotiation (*p* = .*01*) and illegal moves (*p* = .047) predicted poorer friendship quality in children with and without ADHD after six months ADHD/ADHD and ADHD/non-ADHD dyads did not differ in friendship issues
Normand, Ambrosoli [71]						No significant predictors that differentiated children in a car race task Negative affect associated in loss of game in structured play in children with ADHD and were more likely to comment negatively on the abilities of their friends in the free play situation (*p <* .*01*) TD children expressed more frustration regarding their own abilities (*p* = .03)
Normand, Soucisse [72]						Children with ADHD and their invited friends engaged in less co-operative play (*p* < .001), displayed less companionship (*p* < .01), less sensitivity (*p* < .001) ADHD dyads more conflict (*p* < .001), and greater negative affect (*p* < .001) No significant findings between dyads in balance of power and game transitions Results did not vary by age, symptoms, friendship length, composition of dyads
Normand, Mikami [34]						No between group difference in perceptions of positive friendship quality Children with ADHD perceived more positive features than their parents (*p* = .000), friends’ parents (*p* = .000), and fewer negative features than their friends (*p* = .010), their parents (*p* = .000), their friends’ parents (*p* = .000) ADHD-externalising disorders predicted greater negative features and fewer positive features in friendship quality (*p* < .05) Internalising co-morbidities not predictive of friendship quality
Redmond [73]						20% of children with ADHD reported “no close friends” Parents of children with ADHD reported significantly fewer friends and less time with their friends than TD children (*p* = .018) Higher no. of close friends for children with ADHD, significantly correlated to decreased physical bullying (*p* < .05), but not verbal bullying Frequency of contact showed no reduction of physical and verbal bullying
Rokeach and Wiener [74]						Social support significantly increased with age for TD adolescents (*p* < .001) Youth (16–18 years old) with ADHD perceived less social support than youth (13–15 years old) with ADHD Females rated same sex relationships as more supportive than males, irrespective of ADHD status (*p* < .04). Higher social support in same-sex friendships, irrespective of ADHD status, gender and age (*p* < .001) No significant differences in conflict between youth with and without ADHD
Smit, Mikami [75]						Children with ADHD nominated 15% of the classmates as friends More friends were associated with reduced friendship quality (*p* < .05) Children with ADHD with internalising comorbidity, fewer reciprocated friendships and potentially negative quality had greater loneliness (*p* = .053) No significant association for comorbid externalising disorders with loneliness
Timmermanis and Wiener [58]						Youth with ADHD reported significantly less PSS from friends vs. TD (*p* < .01) Female participants had more PSS from friends than male participants (*p* = .03*)* Trend for adolescents with ADHD to perceive less PSS from friends (*p* = .08) No association between bully status and PSS from friends

Note: ADHD = Attention-Deficit Hyperactivity Disorder; No. Number; ODD = Oppositional Defiant Disorder; PSS = Perceived Social Support; TD = Typically Developing

^a^Outcomes were mapped against our 4 domains of friendship based on Bagwell & Schmidt’s [21] and Hartup’s [16] models of friendship as (1) presence of friendships, (2) friendship quality, (3) characteristics of friends, (4) friendship interactions

^b^The role and impact included where friendship was investigated as a correlation, mediator, or moderator on social, emotional and mental health outcomes

### Friendships of children with ADHD

In addressing research question two, the main findings on the friendships of children and youth with ADHD are reported under each friendship domain, with a focus on comparing their friendships to TD children.

#### Domain 1: Presence of friendships

Of the seventeen studies that investigated the *presence of friendships*, thirteen included a TD comparison group. Twelve studies reported children with ADHD had fewer reciprocated friendships compared to TD peers [24, 27, 30–33, 40, 63, 67, 71–73]. Furthermore, every study with a sample of children with ADHD in which reciprocal nominations or parent/teacher corroboration were used in conjunction with the nominations of children with ADHD showed that they have fewer friends [32, 40, 67]. In the two studies without a comparison group, children and youth with ADHD were reported to have none or a small number of friends [55, 75]. Two studies found that children and youth with and without ADHD nominated a similar number of friends [40, 70]. Notably, in one study parents of youth with ADHD reported a similar number of reciprocal friends to TD peers [70], whereas two studies reported that parents of children with ADHD [40] and youth with childhood and persist ADHD reported fewer reciprocated friendships [10]. Friendship stability was investigated in six studies, of which four studies found children with ADHD had shorter friendships than TD peers [24, 30, 32, 40], and one study found that boys with ADHD perceived greater difficulty making friends compared to TD boys [64].

#### Domain 2: Quality of friendships

Eight studies investigated friendship quality, four studies explored one positive feature of friendship quality (PSS from friend, see Table 3). Six of eight studies found children and youth with ADHD self-reported poorer quality friendships, having both less positive features and more negative features compared to TD counterparts [24, 27, 30, 32, 34, 66, 72]. Over six months, Normand, Schneider [24] found both children with ADHD and their invited friends had poorer friendship quality and less satisfaction than comparison dyads. One study reported no differences in friendship quality between youth with and without ADHD [59]. Three studies found that children and youth with ADHD reported perceiving less social support from friends in comparison to a normative sample and their peers [58, 69], with Rokeach and Wiener reporting significantly lower self-reported friendship quality for 16–18 year olds [74]. In a study without a comparison group, children with ADHD reported that PSS was the least available from their friends [68]. On the whole, the studies reviewed indicate children with ADHD report or are observed to have more conflict with their friends, fewer positive features of friendship quality, and lower levels of social support [26, 28–31, 47, 48, 52, 53, 56].

#### Domain 3: Characteristics of friends

Five studies reported on the characteristics of friends. Three studies found that children with ADHD were more likely than TD children to have a friend who also had a learning or behavioural difficulty or also displayed clinical ADHD symptoms [27, 32, 40]. Similarly, friends of youth with ADHD were more likely to have behavioural difficulties or be two years younger or older compared to controls [40, 70]. Adolescent girls with ADHD were less likely to attend the same school as their best friend [70]. Youth with persistent ADHD had more friends who engaged in fewer conventional activities compared to their TD peers [10]

#### Domain 4: Friendship interactions

Six studies investigated the quality of interactions in children with ADHD and their friends [10, 24, 27, 71, 72, 75] and four studies reported the frequency of contact with friends [33, 40, 70, 73]. Four studies found children with ADHD and their invited friends were more likely to have poorer quality friendship interactions than comparison dyads [24, 27, 71, 72, 75] which persisted over six months (regardless of their ADHD status; [24, 27]). There were no significant differences reported for adolescents with and without ADHD in their self-perception of their competence in their friendships [10].

Mixed findings were reported across studies that examined frequency of contact with friends. Three studies demonstrated children with ADHD reported spending less time with their friends outside of school [33, 40, 73]. Conversely, a fourth study found youth with and without ADHD spent a similar amount of time with their friends [70].

#### Friendship outcome measures

Across the twenty-four studies, eleven studies used self-reporting only, five studies used clinician observation and self-reporting, six studies used self- and parent and/or teacher reporting, two studies used parent reporting only and 24 different outcome measures were used (see Table 2). To measure the *presence of friendship*, sociometric peer nominations were most commonly used to determine a reciprocal friendship (*n* = 10) and a non-standardized report to measure friendship stability (*n* = 6). *Friendship quality* was measured by a range of standardized outcome measures, with the Friendship Quality Questionnaire [25] (*n* = 3)and Friendship Quality Measure [83] (*n* = 3) being the most commonly used (see Table 2). To measure the *characteristics of friends*, a parental non-standardized report was most commonly used (*n* = 4). In the domain of *friendship interaction*s, frequency of contact was measured through child and/or parent report (*n* = 4), and the quality of interactions was measured by clinician observation in structured and free play tasks (*n* = 5).

### Friendship, social-emotional wellbeing, and mental health

Regarding research question three, we identified thirteen studies that assessed friendship as a correlating (*n* = 10), mediating (*n* = 1) or moderating (*n* = 2) factor in children’s social-emotional wellbeing and mental health (see Table 3). Aspects of social-emotional wellbeing and mental health measured across the studies were loneliness (*n* = 3), peer victimization (*n* = 4), externalising symptoms/behaviour (*n* = 4), depressive or anxiety symptoms (*n* = 2), affect (*n* = 1), hope (*n* = 1), perceived competence (*n* = 1), self-worth (*n* = 1), sibling relationships (*n* = 1).

Higher quality friendships were reported to be associated with a reduced degree of loneliness for boys with ADHD [64] and youth with ADHD and comorbid learning difficulties [59]. Smit and colleagues [75] found that children who reported negative *friendship quality* also reported to be more lonely although, no significant association between loneliness was found for children with comorbid externalising disorders. Two studies investigated the associations between *friendship quality* and depressive and anxiety symptoms [66, 69]. Only one study found increased *friendship quality* to mediate depressive and anxiety symptoms in adolescents with ADHD [66]. Children with ADHD who had a co-morbid externalising disorder were predicted to have more negative and fewer positive features of friendship quality, however, positive or negative friendship quality was not predicted by a comorbid internalising disorder [34].

In children with ADHD, two studies found that the higher *friendship quality* was correlated with increased overall sense of hope and competence [68] and self-worth [53]. One study reported that increased *friendship quality* was associated with increased positive affect but found no reduction in negative affect [59]. One study investigated the role of *friendship quality* on peer victimization and bully status and found a correlation between victimized youth reporting less PSS from friends [58].

One study investigated the associations between internalising and externalising behaviours and *the presence of friendship* [31]. Only children with comorbid anxiety were reported to have fewer dyadic friends, as this variable was not significant for children with comorbid oppositional defiant disorder-conduct disorder [31]. Three studies produced mixed findings on the associations between *presence of friendship* as a protective function against peer victimization [63, 67, 73]. A negative correlation was reported between numbers of friends and being a victim of bullying [67]; Ma, however, reported that this correlation pertained to boys [73]. Conversely, in girls with ADHD, the *presence of a friend* was reported to reduce peer victimisation as having at least one friend moderated the association between behavioural risk factors (internalising, externalising behaviours and social competence) and victimisation [63]. An association was reported where children with ADHD who had externalizing disorders were also more likely to be bullies themselves if they had more friends [67]. The *presence of friendship* was found to be predicted by quality of sibling relationship in children with ADHD whereas, for TD children, the quality of their sibling relationships could predict both *presence* and *quality of friendship* [32].

### Methodological quality and risk of bias

For the fourth research question, twenty-two studies had strong methodological quality and two studies had good methodological quality [33, 56] (see Table 2). Inter-rater agreement for QualSyst checklist represented strong agreement kw = 0.81 (95% CI = 0.68–0.94), with an average of 14.39 (SD = 1.5) out of 20 items rated as yes on the AXIS. The most common areas where studies needed to report more explicitly to improve their quality score was using a cross-sectional design [10, 17, 27, 30, 32, 33, 58, 59, 63, 64, 67, 69, 70, 73, 74]. There was a risk of selection bias as 14 studies used sampling across one or two locations from referrals and/or self-selection processes. Only three studies justified the sample size using a power analysis [34, 67, 75], with remaining studies reporting small sample sizes. Effect sizes were not completely reported for seven studies.

## Discussion

The aim of this systematic review was to synthesise and methodologically appraise studies reporting on the friendships of children and youth with ADHD. We also compared their friendships to those of TD children and investigated the reported associations between friendship and social-emotional wellbeing and mental health. Nineteen studies were identified, fourteen of which included a TD comparison group. The heterogenous methods of defining, measuring and reporting on friendship across the reviewed studies meant that a collective analysis for making comparisons between the friendships of children and youth with ADHD and TD children was not possible. Previous systematic and literature reviews that have also highlighted difficulties in analysing data from studies using heterogenous friendship measures [4, 8, 17, 29].

Nonetheless, trends across the studies emerged when findings were mapped against the four domains of friendship adopted for this review. Within the friendship domain of *presence of friendships*, children and youth with ADHD were found to have shorter friendships than TD children and young people [24, 27, 30, 40] as well as fewer friends [10, 24, 27, 30–32, 40, 63, 67, 71–73]. Children with ADHD will commonly have difficulties with their social skills and social information processing [7], which may explain their fewer and shorter friendships. One study highlighted how poorer social behaviours, such as noncompliance, aggression, predicted negative peer nominations in boys with ADHD from their first impression [98]. A previous literature review established social-cognitive skills such as sharing, co-operating, attending to social cues, and perspective taking are crucial to successfully initiate and maintain high-quality friendships [28].

Across *friendship quality* and *friendship interactions*, children with ADHD reported or were observed to have more conflict with their friends and fewer positive features to friendship quality [24, 27, 30, 32, 40, 59, 71, 72] including lower levels of PSS [58, 68, 69]. Impairments in social-cognitive functioning may also be contributing to the poorer friendship experience of children with ADHD [39, 42, 99]. For example, social information processing impairments may hinder their ability to attend to a friend as they may miss or misinterpret key social cues, due to either the hostile attribution bias or positive illusionary bias [35, 38, 41]. This means children may respond defensively to others in their interactions and/or lack the insight to reflect on how their behaviour may be negatively impacting others [38, 41]. Further, both Barkley [35] and Cordier and colleagues [39] highlight how poorer *friendship quality* and *friendship interactions* for children with ADHD may be accounted for by difficulties in key emotional and socio-cognitive skills such as emotional regulation, perspective-taking and anticipating the consequences of their actions. Children with ADHD may be more likely to respond inappropriately in conflict or lack empathy to meet their friend’s needs and consequently may lose a friend as a result of their difficulties in emotional regulation and social skills [28, 29, 35, 39, 40, 43]. Furthermore, Heiman reports that children with ADHD often view a close friend as someone who is fun and entertaining, in contrast to TD peers who value emotional support [33]. As companionship is a key component to friendship quality [100], the differences in understanding and values could account for the poorer quality friendships of children with ADHD. This also may explain why difficulties persist into adolescence as TD adolescent friendships are characterised by increased levels of intimacy, self-disclosure, empathy and emotional support [101, 102], whereas adolescents with ADHD may not have had the opportunity to develop these skills in middle childhood by valuing friends that are fun and entertaining [33]. Investigating how aspects of friendship quality vary for children with ADHD across childhood and into adolescence relative to TD children is worthy area of future research. Further, the emotional and socio-cognitive skills above are crucial to developing companionship in childhood as well as adjusting to the increased demands on empathy and emotional support needed for successful friendships in adolescence [17]. The provision of evidence-based interventions that improve children’s socio-cognitive skills across all stages of development may therefore have a positive impact on the quality of friendships and friendship interactions for children with ADHD. Including friends of children with ADHD in those interventions may also increase effectiveness if friends are provided with strategies that support the socio-cognitive skills of children with ADHD.

Every study examining *characteristics of friends* of children and adolescents with ADHD showed that they had more friends with learning and/or behaviour difficulties or ADHD, and more friends who were either two years older or younger [24, 27, 32, 40, 70]. This domain was also the most under-investigated aspect of friendship across the studies. Hartup [16 p6] suggests that the phenomena of similarities among friends has three origins: sociodemographics (similar conditions bring children together), social selection (children choose to become friends with children who are similar, as opposed to children who are different), and mutual socialisation (children who socialise together become more alike). Previous studies have supported the notion that “social selection” can explain the similarities between children with ADHD and their friends, demonstrating how children and youth with ADHD may gravitate towards friends who share similar interests and attitudes [10, 11, 30]. This review was unable to identify strong evidence for the impact of “mutual socialisation” on the characteristics of the friends if children with ADHD. One study reviewed demonstrated how poor quality friendship interactions predicted poorer friendship quality [24], but a scarcity of longitudinal studies limits our ability to understand whether a gradual influence occurs over time between children with ADHD and their friends whereby children’s behaviours become more similar over time. Given that children and youth with ADHD are more likely to select friends with learning or behavioural difficulties with ADHD, the notion of “mutual socialisation” suggests that friendships may also negatively influence their behaviour and increase the risk of maladjustment [10, 11, 30]. Including the friends of children with ADHD in social-cognitive interventions may further increase the intervention’s effect on friendship interactions, as their friends may also benefit from receiving the same socio-cognitive supports.

The nature of friendships for adolescents with ADHD compared to those of TD youth remains relatively unknown. Only three studies reviewed investigated the *presence* and *characteristics of friends* during adolescence, and conflicting evidence was found for *friendship quality* in this age group. Some studies demonstrated that youth with ADHD had poorer *friendship quality*, while others found they experienced similar *friendship quality* as TD youth or no significant differences were found with their self-reported competence. Using parent report to measure the nature of friendships during adolescence may reduce accuracy of findings as the parents of youth (with or without ADHD) may be unaware of their children’s friendships as young people begin spending more time with their friends without parental supervision [70]. Alternatively, similarities in friendships between adolescents with and without ADHD may occur if young people become more tolerant of their friend’s behaviour over time [6]. A recent study examined the perspectives of adolescent youth with ADHD on their friendships where participants had established at least one close friend in late adolescence (16–18 years old) [103]. Participants discussed the developmental impacts of friendship difficulties in middle childhood due to difficulties with social skills and perspective taking and their experiences of peer victimisation, where these skills may have gradually improved over time [103].

However, in this review, no study assessed the *quality of friendship interactions* in youth with ADHD, so we are unable to confirm this explanation. Current evidence suggests future studies are required, to consolidate an understanding of the nature of friendships of youth with ADHD, particularly around the quality of their real-life interactions.

Mixed results were found among studies investigating associations between friendship and social-emotional wellbeing and mental health, echoing findings of previous studies that both support and challenge the role and impact of friendship [21, 25, 30, 104, 105]. While existing research identifies that *presence of friendship* can buffer social and emotional well-being difficulties in children with ADHD [21, 25, 30, 31, 63, 104, 105], our findings indicate that for children with ADHD having friendships may be a protective factors against bullying and victimisation. We found greater evidence to support an association between higher *friendship quality* and positive emotional wellbeing and reduced loneliness. Notably, the heterogeneity among the methods used for measuring friendship across the studies may account for these mixed findings. Consideration of how each friendship domain contributes to social-emotional wellbeing and mental health is required within future research to address the conflicting findings found within current evidence.

Finally, the above findings relating to the friendships of children and youth with ADHD should be interpreted with caution due to limitations in the study design and sampling frames of the included studies. A majority (*n* = 23) of the studies were cross-sectional in their design, limiting our ability to understand how friendships evolved over time. Our findings also may not be generalisable to the breadth of children and youth with ADHD as most studies used small sampling frames with a self-selection and/or referral process.

### Limitations

This study was strengthened by adhering to the PRISMA protocol [47] completing a rigorous search across six databases and implementing two critical appraisal tools to assess methodological quality [56, 57]. Due to the exploratory nature of this systematic review, eighteen included studies used a cross-sectional design. Consequently, our ability to report on the evolving nature of friendship and casual relationships across social and emotional wellbeing outcomes is limited. Further, a secondary reviewer was unable to assess 50% overlap using AXIS due to time constraints. No meta-analysis was conducted due to the clinical heterogeneity seen in variable friendship measures, and small sample sizes.

### Implications for research and clinical practice

We propose five areas for further investigation. Firstly, there is a need for longitudinal studies using the real-life friendships of children and youth with ADHD. This will enable greater insights into how the friendship domains may evolve over time in conjunction with social, emotional and cognitive development. Secondly, future studies should assess the mediating and moderating roles of the four friendship domains in the prevention of social, emotional and mental health difficulties. We propose that further studies should also investigate how underlying capacities, including social-cognitive skills and emotional regulation skills, contribute to the poorer friendship experience across the friendship domains of children and adolescents with ADHD. By examining these relationships, this could inform existing interventions, such as Children’s Friendship Training [106], Program for Evaluation and Enrichment of Relational Skills (PEERS) for adolescents [107] or classroom based interventions such as the Making Social Accepting Inclusive Classrooms (MOSAIC), and novel friendship interventions to target the underlying capacity (i.e. emotional regulation) in a naturalistic setting (i.e. with friends or classmates). We also identified a range of self-reporting, parent reporting and clinical observation measures to assess our friendship domains. We recommend that future studies assess the psychometric properties of these tools to compare their usefulness for future studies. Finally, as fourteen of the studies were conducted in English speaking countries, the results in the review reflect a limited cultural perspective of friendships. Therefore, cross-cultural comparisons of friendships should be considered in future studies to understand how cultural values may impact children and youth’s perspective and experience of friendship.

Clinically, by synthesising current literature in our four domains, clinicians can use these domains to explore a client’s strengths and weaknesses in their friendships more effectively. Thus, children and youth with ADHD may experience greater success in their friendships and prevent the negative impacts on their development and well-being.

## Conclusion

This systematic review has demonstrated that children with ADHD have poorer friendships compared to TD peers, particularly with respect to the presence of friendships and friendship quality. There is limited evidence on the friendships of youth with ADHD and conflicting evidence for the associations between friendship and social-emotional wellbeing and mental health. The risk of bias within studies and cross-sectional nature of their design mean that findings should be interpreted with caution and cannot be generalised to all children with ADHD. Future research should assess the nature of children’s friendships over time, the buffering roles of friendships, and the psychometric properties of the friendship measures.

## Supporting information

S1 Checklist PRISMA 2020 checklist. (PDF)
